# Recruitment for Health Disparities Preventive Intervention Trials: The Early Childhood Caries Collaborating Centers

**DOI:** 10.5888/pcd11.140140

**Published:** 2014-08-07

**Authors:** Tamanna Tiwari, Alana Casciello, Stuart A. Gansky, Michelle Henshaw, Francisco Ramos-Gomez, Margaret Rasmussen, Raul I. Garcia, Judith Albino, Terrence S. Batliner, The Early Childhood Caries Collaborating Centers

**Affiliations:** Author Affiliations: Alana Casciello, Michelle Henshaw, Raul Garcia, Boston University Henry M. Goldman School of Dental Medicine, Boston, Massachusetts; Stuart A. Gansky, Margaret Rasmussen, University of California San Francisco, School of Dentistry, San Francisco, California; Francisco Ramos-Gomez, University of California Los Angeles, School of Dentistry, Los Angeles, California; Judith Albino, Terrence S. Batliner, Centers for American Indian and Alaska Native Health, Colorado School of Public Health, University of Colorado Anschutz Medical Campus, Aurora, Colorado; The Early Childhood Caries Collaborating Centers.

## Abstract

**Background:**

Four trials of interventions designed to prevent early childhood caries are using community-engagement strategies to improve recruitment of low-income, racial/ethnic minority participants. The trials are being implemented by 3 centers funded by the National Institute of Dental and Craniofacial Research and known as the Early Childhood Caries Collaborating Centers (EC4): the Center for Native Oral Health Research at the University of Colorado, the Center to Address Disparities in Children’s Oral Health at the University of California San Francisco, and the Center for Research to Evaluate and Eliminate Dental Disparities at Boston University.

**Community Context:**

The community contexts for the EC4 trials include urban public housing developments, Hispanic communities near the US–Mexican border, and rural American Indian reservations. These communities have a high prevalence of early childhood caries, suggesting the need for effective, culturally acceptable interventions.

**Methods:**

Each center’s intervention(s) used community-based participatory research approaches, identified community partners, engaged the community through various means, and developed communication strategies to enhance recruitment.

**Outcome:**

All 3 centers have completed recruitment. Each center implemented several new strategies and approaches to enhance recruitment efforts, such as introducing new communication techniques, using media such as radio and newspapers to spread awareness about the studies, and hosting community gatherings.

**Interpretation:**

Using multiple strategies that build trust in the community, are sensitive to cultural norms, and are adaptable to the community environment can enhance recruitment in underserved communities.

## Background

The objective of this case study was to examine community engagement efforts to enhance recruitment for the 3 Centers for Research to Reduce Oral Health Disparities (CRROHD) that comprise the Early Childhood Caries Collaborating Centers (EC4) funded by the National Institute of Dental and Craniofacial Research (NIDCR): the Center for Native Oral Health Research (CNOHR) at the University of Colorado, the Center to Address Disparities in Children’s Oral Health (CANDO) at the University of California San Francisco, and the Center for Research to Evaluate and Eliminate Dental Disparities (CREEDD) at Boston University.

These centers develop and test interventions for preventing early childhood caries among populations that have disparities in disease burden and use a community-based participatory research (CBPR) approach. A CBPR approach increases the value of research for the community and researchers; it creates a bridge between the two (1), establishes mutual trust, and develops culturally appropriate interventions (2).

NIDCR recognizes that successful interventions in racial/ethnic minority communities require CBPR approaches. Therefore, the funding opportunity announcement (http://grants.nih.gov/grants/guide/rfa-files/RFA-DE-99-003.html and http://grants.nih.gov/grants/guide/rfa-files/RFA-DE-08-008.html) for CRROHD explicitly required applicants to develop interventions with CBPR approaches and demonstrate participation by communities in the proposed interventions. Following these guidelines, each center submitted detailed plans outlining engagement of community advisory boards (CABs) that meaningfully involved community partners and communication strategies to enhance recruitment of and interaction with study participants.

## Community Context

The 3 community contexts of the EC4 studies vary considerably — urban public housing developments in Boston, Hispanic communities near the US–Mexican border, and rural American Indian reservations. Residents in Boston public housing developments are low-income families; 78% of households have annual incomes below $20,000; 50% are Hispanic, 32% black, 10% white, and 8% Asian (3). In the US–Mexican border communities, 83% of the population is Hispanic and 30% lives in poverty. The participating American Indian populations live in remote, rural clusters on reservations; their poverty rate is 29%, and they have significant health inequalities (4).

Each community has a high prevalence of early childhood caries. Children who have early childhood caries may experience pain, difficulty chewing, problems sleeping, trouble concentrating, missed school days, fatigue, irritability, depressive symptoms, behavioral issues, reduced self-esteem, and even reluctance to smile or laugh (5,6). Early childhood caries can also cause early tooth loss, which may affect speech development, nutrition, and permanent tooth eruption patterns (7).

The prevalence of untreated caries among American Indian and Alaska Native children aged 2 to 5 years is 43.6% (8). Baseline data from CNOHR Study II found that 69.5% of Southwestern tribal children aged 3 to 5 years have untreated decay (9). Nationally in 1999–2004 among children aged 2 to 4 years, the prevalence of untreated caries was 35.5% among Mexican Americans and 20.5% among non-Hispanic whites. Among poor children aged 2 to 4 years, the prevalence was 43.8% among Mexican Americans and 34.7% among non-Hispanic whites (10). The 3-year incidence of caries was 32.5% among 3-year-olds in the control group of a study in the border community (11).

Mistrust of researchers, government, and academic institutions is a central barrier to recruitment of racial/ethnic minority populations (12), especially in the American Indian community (13). Fear of mistreatment and exploitation, language barriers, lack of adequate information about research, and time and financial constraints also pose difficulties in recruiting some minority groups (12,14,15). Investigators at the 3 centers asserted that these barriers could be overcome by community–academic partnership and communication with community partners to establish concordance between community and center goals.

The objective of the community–academic partnerships was to develop and test community-based and culturally sensitive preventive interventions to reduce early childhood caries among young children in low-income and racial/ethnic minority populations.

## Methods

The EC4 formed several collaborative working groups to develop and share information on methods to ensure that results of each center’s studies would be comparable and generalizable. One working group, the EC4 Recruitment and Retention Working Group, holds periodic teleconferences to share information, discuss common problems, and explore innovative methods for participant recruitment and retention. Each center received approval from NIDCR staff and the center’s institutional review board. CNOHR also received approval from tribal review boards.

CNOHR has 2 culturally tailored; randomized controlled trials of behavioral interventions in 2 reservation locations (Table 1). Promoting Behavior Change for Oral Health in American Indian Mothers and Children (CNOHR Study I) (ClinicalTrials.gov: NCT01116726) assesses the efficacy of a culturally tailored, motivational interviewing intervention to prevent caries in children through knowledge and behavior change in new mothers (16). The intervention is delivered by trained community members.

**Table 1:** Early Childhood Caries Collaborating Centers, Research Foci, Target Populations, Recruitment Sites and Age of Participants Recruited

University Affiliation	Center	Study	Age of Children at Recruitment	Recruitment Sites
University of Colorado	Center for Native Oral Health Research	Study I	0–3 mo	Indian Health Service hospitals, WIC clinics, and Native Women’s Health clinics
		Study II	3–5 y	Head Start centers
University of California San Francisco	Center to Address Disparities in Children’s Oral Health	Glass Ionomer Sealant and Fluoride Varnish Trial	2.5–3 y	WIC clinics, Head Start programs, health clinics, and daycare centers
Boston University	Center for Research to Evaluate and Eliminate Dental Disparities	Tooth Smart Healthy Start	0–5 y	Public housing developments

Abbreviation: WIC, Special Supplemental Nutrition Program for Women, Infants, and Children.

Preventing Caries in Preschoolers: Testing a Unique Service Delivery Model in American Indian Head Start Centers (CNOHR Study II) (ClinicalTrials.gov: NCT01116739) is a community-based trial in 52 Head Start programs for a single southwestern tribe (17). The intervention is delivered by community oral health specialists who are lay tribal members who have received brief training from oral health and behavioral experts.

The theme of CANDO is to understand, prevent, and reduce oral health disparities among young children, focusing primarily on preventing early childhood caries. The Glass Ionomer Sealant and Fluoride Varnish Trial (GIFVT) (ClinicalTrials.gov: NCT01129440) is a stratified, randomized trial to test fluoride varnish alone versus fluoride varnish plus fluoride containing glass ionomer sealants on posterior occlusal (chewing) primary tooth surfaces (Table 1). The intervention is delivered in community health centers near the US–Mexico border. One study aim is to assess parental acceptance of the interventions and factors related to lost-to-follow-up.

The organizing theme of CREEDD is engaging non-dental care providers in oral health promotion and extending venues for oral health promotion to nonclinical care settings. The Oral Health Advocates in Public Housing project (ClinicalTrials.gov: NCT01205971), which community representatives renamed Tooth Smart Healthy Start (TSHS), is a stratified cluster-randomized trial in 26 public housing developments in Boston (Table 1). All participants receive oral health assessments, feedback on oral health status, and fluoride varnish. In addition, participants at experiment sites complete motivational interviewing sessions with oral health advocates who are lay health workers trained in motivational interviewing and oral health education.

### Community engagement efforts to enhance recruitment

**Academic–community partnership. **Community partners are involved in developing and implementing the interventions as part of a CBPR approach. Each center has one or more active CABs, which involve stakeholders from various arenas including education, health care, childcare, public health, local churches, and the general community. The objectives of each CAB are to shape the intervention, advocate for the community’s participation, and advise study investigators on recruitment strategies and other study procedures. Each center meets with its CABs at least twice a year to report on study progress and discuss methods for enhancing community participation.

Each CAB reviewed the cultural appropriateness of recruitment materials, including posters, flyers, brochures, and radio scripts. Both CNOHR and CREEDD relied on advice from their community/tribal members when designing the posters, flyers, and logos; CNOHR translated study titles into tribal language in response to a request by its CAB.

Each center and its CAB discussed participant compensation amounts (distributed only to enrolled participants) and determined those amounts according to community norms or tribal policies. Each CAB will assist in interpreting and disseminating results at study conclusion. CNOHR was challenged by tribal values when its study design initially included an assessment-only control group. Tribal representatives viewed the study design as withholding treatment from some participants; the tribal communities required that all participants receive some benefit. In response, CNOHR created “enhanced community service groups” (instead of control groups) in which participating children received basic oral health information, toothpaste, and toothbrushes.

The GIFVT study grew out of the CAB’s recommendation to test ways to prevent caries in toddlers who had participated in CANDO’s previous study.

The TSHS team conducted focus groups with caregivers in several housing developments to gather information about knowledge, attitudes, and beliefs about children’s oral health and to solicit input on a study design to improve children’s oral health. Community input was also obtained through meetings with the property manager and tenant task force representatives of each housing development to ascertain recruitment strategies used in other research or community/social service programs. This proactive solicitation of community input ensured greater cultural and social relevance to the population served and likely improved recruitment.

**Community engagement. **Centers engaged the community through participation by study staff in community and social events, health fairs, and other cultural activities. Not all centers successfully recruited participants through these activities, but the activities were resource-efficient venues through which community connections were maintained.

Providing culturally appropriate food at community events and gatherings is highly valued in many communities as a gesture that elicits and builds trust. Because of federal funding policies, however, the ability to provide food at events was sometimes limited. Several principal investigators used discretionary or personal funds for food at recruitment events when advised by community members that not to do so would create a negative impression.

**Communication. **All studies strategically placed posters or flyers to increase awareness of study opportunities. The main communication tool, apart from in-person contact with participants, was the telephone. Disconnected or nonfunctioning telephone lines posed one of the most common and difficult challenges. Some studies found that one telephone line was shared among several people, or telephone access was borrowed from a family member or neighbor. Some potential participants did not have landline service and relied on prepaid cellular service requiring careful allocation of minutes. All 3 centers requested telephone numbers of family, friends, or neighbors and mailed or hand-delivered postcards as other strategies to overcome temporarily nonworking telephone service. TSHS included its recruitment flyer in the rent statement and newsletters of the housing developments; however this strategy was not as effective as door-to-door recruitment.

CNOHR Study I asked study participants for their opinions on telephone use and costs. Some participants preferred text messaging to telephone calls and others preferred Facebook. Facebook accounts do not require a monthly payment and can be accessed through various means, including free public libraries that proved a stable and reliable communication mode for many participants. However, confidentiality considerations make it unsuitable in certain settings. Based on participant responses, CNOHR Study I provided texting and Facebook messages as alternative communication tools.

## Outcome

### Planned recruitment and present recruitment numbers

The CNOHR Study I enrollment goal was 600 American Indian caregiver–child dyads; enrollment began in September 2011 and was completed in February 2014 after estimated contact with 1,461 individuals. CNOHR Study II contacted 82 Head Start centers and recruited 52 centers, which were randomized into intervention and control groups (26 per group); 1,016 American Indian participants were then enrolled from the randomized Head Start centers during 2 years (2011 and 2012), with 562 enrolled at the beginning of the first school year and 454 at the beginning of the second.

GIFVT’s enrollment goal was 596 caregiver–child dyads within 20 months. Recruitment began in June 2011 and ended in January 2013. The sample size of 597 randomizations was reached in 19 months after an estimated 1,321 contacts with 1,158 individuals.

TSHS enrolled 1,421 caregivers with children younger than 6 years from 26 public housing clusters. Recruitment began in January 2011 and ended in March 2014 after estimated contact with 2,876 individuals.

### Strategies to enhance engagement of the community

Each center implemented several new strategies to enhance recruitment efforts (Table 2).

**Table 2:** New Strategies Used by Early Childhood Caries Collaborating Centers to Enhance Recruitment of Participants for Interventions to Prevent Early Childhood Caries

New Strategies	Center for Native Oral Health Research		Center to Address Disparities in Children’s Oral Health — Glass Ionomer Sealant and Fluoride Varnish Trial	Center for Research to Evaluate and Eliminate Dental Disparities — Tooth Smart Healthy Start
	Study I	Study II		
Advice from community advisory board and community members	X	X	X	X
Social media (Facebook)	X			
New communication techniques (e-mail, text messaging, postcards)	X		X	X
Radio public service announcements	X			
Snowballing/refer-a-friend	X		X	
Community host gathering			X	X

**Figure 1:**
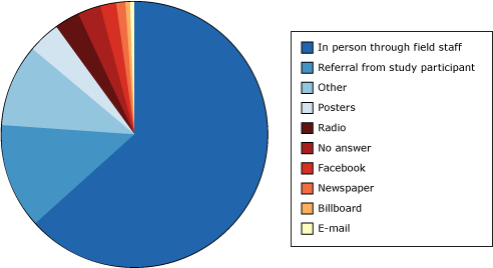
How participants heard about the Center for Native Oral Health Research Study I (N = 297). **Table:** 

Despite CNOHR Study I’s slow start, participation improved by expanding the study to include tribal members living outside yet near the reservation, adding local staff, and recruiting at community activities such as Pow Wows, basketball tournaments, and health fairs. New communication methods were introduced, including texting, public service announcements on local radio, and a Facebook page. CNOHR Study I collected data on the effectiveness of these new communication strategies for participants enrolled after September 2012 (n = 297) (Figure 1): 64% of participants heard about the study through field staff at health care centers and other locations; 2% through Facebook; 12% through other study participants; and 8% through posters, radio public service announcements, newspaper advertisements, and billboards.

How did you hear about us?	No. of Participants (N = 297)	Percentage
In person through field staff	191	64
Referral from study participant	37	12
Other	31	10
Posters	11	4
Radio	8	3
No answer	8	3
Facebook	6	2
Newspaper	3	1
Billboard	1	<1
E-mail	1	<1

CNOHR Study II benefited from close collaboration between Head Start teachers and field staff. Field staff visited each classroom to introduce the study and made visits at the beginning and end of each enrollment year. Enrollment teams assigned to classrooms based on geographic location offered two 8-week enrollment opportunities each year. Head Start teachers and staff sent study information to all families and actively promoted recruitment through their daily contact with families. Support from Head Start teachers and from local and tribal administrators enhanced enrollment.

**Figure 2:**
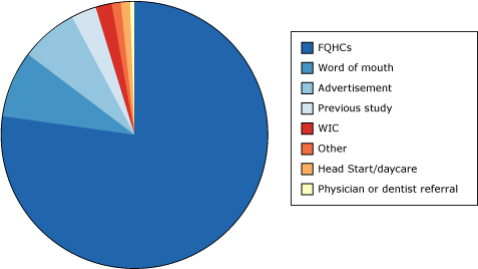
How participants heard about the Glass Ionomer Sealant and Fluoride Varnish Trial study (N = 604 responses). Abbreviations: FQHCs, federally qualified health centers; WIC, Special Supplemental Nutrition Program for Women, Infants, and Children. **Table:** 

GIFVT staff recruited about three-quarters of its participants in pharmacies and adult and pediatric medical and dental waiting rooms at federally qualified health centers (Figure 2). GIFVT recruited a substantial number of participants through word of mouth and advertisements. Although GIFVT investigators originally estimated that about one-third of participants could be recruited from a previous study, many of those participants no longer met the age criterion after delays caused by additional regulatory requirements; consequently, only 3% of enrollees were recruited from the previous study.

How did you hear about us?	No. of Responses (N = 604)	Percentage^a^
FQHCs	462	77
Word of mouth	51	8
Advertisement	45	7
Previous study	16	3
WIC	11	2
Other	9	1
Head Start/daycare	7	1
Physician or dentist referral	3	<1

Abbreviations: FQHCs, federally qualified health centers; WIC, Special Supplemental Nutrition Program for Women, Infants, and Children.

^a^ Percentages do not add to 100% because of rounding.

Because so few of the previous study participants were eligible for the GIFVT study, the CAB suggested recruiting their younger siblings, cousins, and neighbors. About 5% of participants named more than one way they heard about the study. Bilingual, culturally sensitive staff played a key role in establishing relationships with participants, a process that was critical to gaining trust and securing enrollment. Knowledge and experience from the previous study and continued commitment to the community informed the team’s recruitment strategies. Staff demonstrated cultural values such as *personalismo*, which fosters warm, friendly, and informal interpersonal relationships, and *familismo* which promotes close, cooperative, and cohesive relationships with extended family and close friends (18). GIFVT staff members, especially the community outreach workers, welcome potential and enrolled participants warmly with personal greetings; explain that the study aims to find ways to improve the health of their children and future children in their families; and at subsequent visits ask participants about family and life events in a way that is considered caring, not prying.

**Figure 3:**
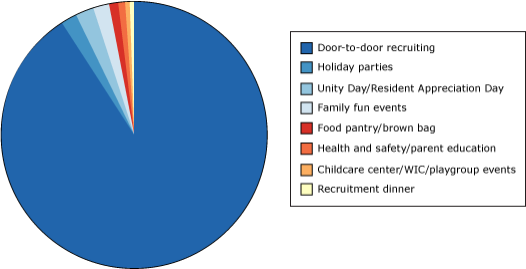
How participants heard about the Tooth Smart Healthy Start study (N = 1,421). No participants were recruited through Bingo or movie nights, so this category is not included in the figure. Abbreviation: WIC, Special Supplemental Nutrition Program for Women, Infants, and Children. **Table:** 

The bilingual TSHS study team capitalized on existing strong social networks in public housing to reach residents. Creating multiple community outreach worker positions bolstered monthly enrollment; these outreach workers focused solely on recruitment instead of splitting efforts between recruitment and data collection. Resource-intensive, door-to-door recruitment was the primary and most successful recruitment strategy used (Figure 3). The research staff attended 134 community events, including 30 resident appreciation celebration barbeques, 26 family fun events, 22 holiday parties, 19 health fairs/parent education sessions, 16 food pantry distribution events, 11 childcare/playgroups, 9 bingo/movie nights, and 1 recruitment dinner. Of the 420 families who provided contact information at these events, 114 were eligible and enrolled in the study (8% of total enrollment).

How did you hear about us?	No. of Participants (N = 1,421)	Percentage
Door-to-door recruiting	1,307	92
Holiday parties	30	2
Unity Day/Resident Appreciation Day	27	2
Family fun events	21	2
Food pantry/Brown Bag	18	1
Health and safety/parent education	9	1
Childcare center/WIC/playgroup events	5	<1
Recruitment dinner	4	<1
Bingo/movie nights	0	0

Abbreviation: WIC, Special Supplemental Nutrition Program for Women, Infants, and Children.

### Common challenges for the centers

Recruiting and retaining study staff, who were hired mostly from the study communities, was a challenge for each study. Because of frequent staff turnover, more time and effort is required to train new staff, which can negatively affect budgets. The TSHS team has used both undergraduate and graduate student interns and volunteers as well as temporary community outreach workers to ensure study progress during periods of staff turnover. The position for the GIFVT community outreach coordinator became vacant during recruitment; during the several months it took to fill the position the remaining study team members helped to fulfill those duties. To retain study team members, all principal investigators encouraged and worked to motivate them, recognized their accomplishments, listened to their concerns and suggestions, and acknowledged their hard work.

Participants sometimes miss initial study enrollment appointments. Participants miss appointments for many reasons, including work schedules, children’s schedules, childcare arrangements, transportation delays, weather conditions, and long commutes to the field office. All studies offered flexible appointment scheduling and provided appointment reminders to improve recruitment. CNOHR Study I and TSHS offered home visits, a successful recruitment strategy. Although convenient for the participants because it reduced their travel time, home visits were challenging for the research staff because it increased travel time and costs. GIFVT offered participants assistance with transportation.

Weather conditions (eg, blizzards, ice storms, heat waves, brush fires); remote residential locations, travel time; road conditions, renovations, and construction at study sites; and personal safety caused cancelation of recruitment events and posed additional barriers to recruitment. The physical environment posed problems for all of the centers, but each location had its unique challenges, and these were perhaps the hardest to solve. GIFVT was able to wait out its extreme weather conditions. To overcome their challenges, CNOHR Study I and TSHS expanded their field staff, hiring from the local community and dividing study teams by recruitment site. Whereas CNOHR Study I assigned teams by recruitment site; TSHS staff members were assigned to study sites according to their availability on the given day and their familiarity with the site.

## Interpretation

Racial and ethnic minority populations are underrepresented in clinical research. Barriers to their participation may include researchers’ lack of cultural knowledge, few evidence-based strategies for community engagement, mistrust of researchers among community members, and logistical concerns (15). While implementing the EC4 studies, investigators encountered several such barriers; however, because of EC4 collaborative efforts and a CBPR approach, each center developed community-engagement strategies that relied on local people’s insights into their cultures and lifestyles. The understanding gained from the academic–community partnership guided research, increased interaction between researchers and community members, and enhanced opportunities for recruitment. Involving community stakeholders in as many venues as possible contributed to building trust in the community. Other strategies adopted were sensitivity to community or tribal norms, flexibility in appointment scheduling, adapting engagement approaches according to community or tribal feedback, and responding to logistical concerns, such as providing transportation to participants and food at social events.

Methods for community engagement vary according to the values, needs, and previous research experience of the community. The most effective community-based research recruitment strategies are open to establishing true community partnerships and learning how best to serve the community.
