# Erratum, Vol. 11, August 7 Release

**DOI:** 10.5888/pcd11.140140e

**Published:** 2014-09-04

The academic degree for author Stuart A. Gansky in the byline of the article “Recruitment for Health Disparities Preventive Intervention Trials: The Early Childhood Caries Collaborating Centers,” was corrected to DrPH. Also, because of a technical error, the second half of the sentence in the Interpretation section of the Abstract was omitted in the HTML version of the article. The full sentence is “Using multiple strategies that build trust in the community, are sensitive to cultural norms, and are adaptable to the community environment can enhance recruitment in underserved communities.”

The corrections were made to our website on August 13, 2014, and appear online at http://www.cdc.gov/pcd/issues/2014/14_0140.htm. We regret any confusion or inconvenience these errors may have caused.

